# Synthesis and Characterization of a Strontium–Quercetin Complex and Its In Vitro and In Vivo Potential for Application in Bone Regeneration

**DOI:** 10.1021/acsomega.4c09949

**Published:** 2025-01-30

**Authors:** Israel B. Pimenta, Gildácio Chaves Filho, Elias G. B. Silva, Lucas F. B. Nogueira, Tomaz Santana de Mendonça, Taíssa C. S. Furtado, Paulo Cesar Ferreira GasparNeto, Luis Gustavo Dias, Sandra Yasuyo Fukada, Pietro Ciancaglini, Ana Paula Ramos

**Affiliations:** † Department of Chemistry, Faculty of Philosophy, Science and Letters at Ribeirão Preto, University of São Paulo, Ribeirão Preto 14040-901, São Paulo, Brazil; ‡ Department of Biomolecular Sciences, School of Pharmaceutical Sciences of Ribeirao Preto, University of Sao Paulo, Ribeirão Preto 14040-903, São Paulo, Brazil

## Abstract

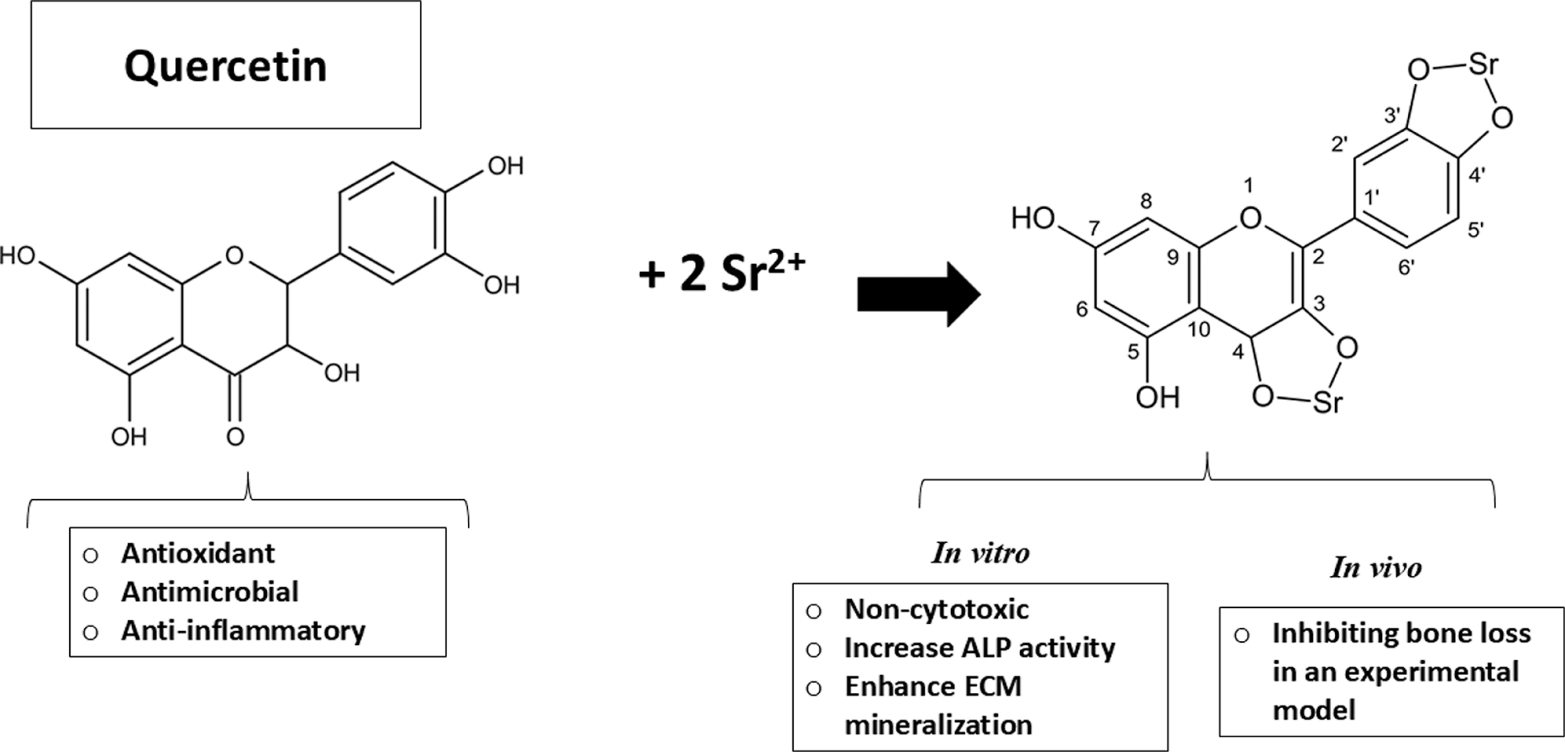

Progress has been made toward developing therapies to treat bone-related diseases and defects caused by trauma. However, some of these therapies, such as administering strontium ranelate to treat osteoporosis, have significant side effects. In this context, designing new and safer strontium-based materials constitutes an important current challenge. Here, we have used quercetin as a platform to synthesize a new complex based on strontium and evaluate its activity in vitro and in vivo. First, we carried out strontium complexation with quercetin. Then, we employed Fourier transform infrared spectroscopy, nuclear magnetic resonance, and thermal gravimetric analysis to determine the chemical composition of the resulting complex as [(C_15_H_7_O_7_)Sr_2_]·6(H_2_O), which was also supported by theoretical calculations. This complex enhanced osteogenic differentiation of a preosteoblastic cell line in vitro, which increased alkaline phosphatase activity and extracellular matrix mineralization. By using a periapical lesion model in mice, we tested whether treatment with this complex could regenerate bone defects in vivo and found that the lesions decreased after 7 days. Together, our data showed that the strontium–quercetin complex synthesized herein is a potential candidate for developing new bone regeneration therapies.

## 1 Introduction

Bone-related diseases and defects resulting from trauma, cancer, infections, and degenerative and inflammatory conditions are increasing on a global scale and have considerably impacted the human health and quality of life.^1−3^ Although bone comprises a dynamic structure that can self-regenerate, pathological conditions or defect size can hinder self-regeneration.^4,5^ The two most common clinical scenarios involving bone homeostasis loss are related to reduced bone density (i.e., osteoporosis and osteopenia) and local trauma (i.e., fractures).^6^ In these situations, bones may fail to self-heal, delaying recovery or causing nonunion (e.g., large segmental defects due to trauma or cancer) regeneration.^5,7^ Deeply understanding clinical risks and biological pathologies is essential when developing effective treatment and bone regeneration strategies.

Osteoarthritis and osteoporosis have commonly been treated with strontium ranelate (SrR), a strontium-based drug that helps regulate bone mineralization.^8^ This drug has been widely applied on the basis of the well-known effects of strontium on bone metabolism—strontium guides bone formation by increasing osteoblast activity and inhibiting osteoclast action.^8,9^ However, the use of SrR has been discontinued due to cardiac concerns^10^ and other side effects like diarrhea, nausea, risk of blood clots, seizures, and memory and consciousness loss.^8,10,11^ Such side effects can be related to the high SrR doses that are needed to achieve positive clinical effects but which may also result in pathological calcification.^12^ These issues notwithstanding, several studies have demonstrated that strontium benefits osteoblast differentiation and mineralization.^13−16^ Furthermore, low strontium doses have been described to affect the bone mineral phase structure positively.^17^ In this context, searching for new strontium-based complexes that can elicit positive responses at low strontium doses has emerged as an alternative to designing new therapies for bone-related diseases. Binding and delivering strontium through biological molecules can be a promising strategy that takes advantage of the beneficial effects of natural compounds.^18−20^ For instance, strontium complexation with biological molecules, such as flavonoids, to achieve positive effects at low strontium concentrations could result in potential new drug candidates.^20^

Flavonoids, a class of natural polyphenolic compounds displaying antioxidant and anti-inflammatory properties, play an essential role in human health.^21−23^ Quercetin (3,3′,4′,5,7-pentahydroxyflavone), a natural flavonoid, promotes osteogenic differentiation of different cell lines.^23−26^ In a recent study, our group described how a novel strontium–morin complex impacts the way osteoblasts and osteoclasts perform in vitro.^20^ Flavonoids can also easily form complexes with metal ions such as aluminum,^27,28^ calcium,^29^ and lanthanides.^30^ Moreover, metal complexes with quercetin have been described to exhibit potential biological activity.^21,31^

Quercetin has been complexed with various metal ions, such as magnesium,^32^ copper,^33^ zinc,^34^ and calcium,^35^ which can modulate its solubility, stability, and bioavailability. For instance, quercetin–copper complexes have demonstrated superior antioxidant and free radical-scavenging capabilities compared to quercetin alone owing to the synergistic effects between the flavonoid and the redox potential of the metal.^33^ These complexes have been explored in oxidative-stress-related diseases, showing promise in reducing reactive oxygen species and mitigating cellular damage. Similarly, quercetin–magnesium complexes have been linked to anti-inflammatory effects by modulating key inflammatory pathways, such as inhibiting the release of pro-inflammatory cytokines like IL-6 and TNF-α.^32,35^ Beyond these effects, quercetin–metal complexes have shown significant potential in bone-related therapies. The quercetin–copper complex, for example, has been reported to enhance osteoblast differentiation and mineralization, possibly by upregulating osteogenic markers such as RUNX2 and ALP.^36^ These findings suggest that the complexation of quercetin with metals amplifies its intrinsic properties and introduces novel biological interactions, paving the way for the development of advanced therapeutics for inflammatory diseases, bone repair, and beyond. Continued research into the bioactivity and mechanisms of quercetin–metal complexes is essential for translating these findings into clinical applications. Nevertheless, the complexation of strontium with quercetin has never been described, which makes our study unprecedented.

Therefore, here, we have used quercetin, a flavonoid with well-described antioxidant,^23,32^ anti-inflammatory, and osteogenic action,^23,24,37,38^ to synthesize new platforms to carry strontium. We have investigated whether quercetin can bind to strontium and evaluated how a new strontium–flavonoid complex affects osteogenic differentiation of a preosteoblast cell lineage in vitro and pathological bone loss in vivo.

## 2 Materials and Methods

### 2.1 Synthesis of the Strontium–Quercetin Complex

The strontium–quercetin complex (SrQ) was prepared by dissolving 0.151 g of quercetin (Sigma-Aldrich-USA, Q4951) in 50 mL of methanol containing 100 μL of NH_4_OH solution (Dinâmica-Brazil, 18–30% NH_3_). Next, 0.800 g of solid SrCl_2_ (Exôdo Cientfica-Brazil) was slowly added to this solution, which was then kept stirring for 2 h. A brownish product emerged, indicating that SrQ was formed. The precipitate was isolated by centrifugation, washed with chloroform/*t*-butanol solution (50 vol %), and dried at 37 °C.

### 2.2 SrQ Chemical Characterization

#### 2.2.1 UV–Vis Analysis

The pure quercetin and SrQ UV–vis absorption spectra were obtained on a Hewlett-Packard model 8453 spectrophotometer. The samples were placed in a quartz cuvette (path length of 1.00 cm) at 25 °C, and methanol was used as a solvent.

#### 2.2.2 Infrared Analysis

Infrared spectroscopy was performed to investigate the SrQ structure. The spectra were acquired on a Shimadzu Prestige 21 IR spectrophotometer operating from 400 to 4000 cm^–1^ with a resolution of 2 cm^–1^. The samples were mixed with KBr (analytical grade) to prepare pellets before the analyses.

#### 2.2.3 Nuclear Magnetic Resonance

^1^H nuclear magnetic resonance (NMR) spectra were obtained on a Bruker Avanca DRX-MHz spectrometer. The samples were dissolved in deuterated dimethyl sulfoxide (DMSO) for analysis.

#### 2.2.4 Elemental Analysis

Carbon and hydrogen elemental analyses were performed on a PerkinElmer 2400 series II analyzer equipped with a thermal conductivity detector. The samples were placed under an O_2_ atmosphere, which was followed by combustion and quantification of the resulting combustion products.

#### 2.2.5 Thermogravimetric Analysis

Thermogravimetric analysis (TGA) was performed on a thermogravimetric analyzer SDT Q600 (TA Instruments). The curves were recorded under air, from 40 to 800 °C, at a heating rate of 10 °C/min. An initial mass of 4.319 mg of SrQ was used.

#### 2.2.6 Conductivity

Conductivity analyses were performed using a conductometer (Methohm 712); SrQ was solubilized in methanol (1 × 10^–3^ M).

### 2.3 Structure Stability: Computational Methodology

SrQ stability was evaluated by using a thermodynamic cycle. This approach involved determining the Gibbs free energy of the gas-phase reaction between the triple charged quercetin anion and two strontium ions. The calculation included the Gibbs free energy differences associated with the transfer from the gas phase to methanol for the three species (SrQ, quercetin anion, and strontium ion). This process is summarized in eq 1
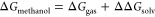
1
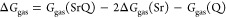

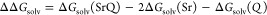
where Δ*G*_methanol_ is the Gibbs free energy for SrQ complexation in methanol; Δ*G*_gas_ is the Gibbs free energy for the gas-phase reaction; ΔΔ*G*_solv_ is the Gibbs free energy difference associated with the transfer from the gas phase to methanol; and *G*_gas_ is the sum of electronic, translational, rotational, and vibrational contributions to the gas phase free energy of an ion. Δ*G*_methanol_ calculation is simplified by assuming that Δ*G*_gas_ can be approximated by the electronic contributions to *G*_gas_, and the solvation free energies can be calculated by the SMD continuum solvent model.

The gas-phase ions were fully relaxed using the B3LYP-D3(BJ)/def2-TZVP method. Tight convergence criteria were applied for both SCF cycles and geometry optimizations. To enhance the computational efficiency, an auxiliary basis set (def2/J) was employed within the resolution-of-identity framework. For the strontium ion, an effective core potential representing 28 core electrons was used, as obtained from the Stuttgart/Cologne pseudopotential library. Following the gas-phase optimizations, Δ*G*_solv_ was computed through single-point calculations at the B3LYP/def2-SVP level. The conductor polarizable continuum model and cavitation–dispersion-specific terms from the SMD model in methanol were incorporated.

All the electronic structure calculations were carried out by using Orca 4.2.1^39^ and Multiwfn 3.8.^40^

### 2.4 In Vitro Experiments

#### 2.4.1 Cell Culture and Maintenance

The murine preosteoblast lineage MC3T3-E1 Subclone 14 (ATCC CRL-2594) was used to study the SrQ osteogenic potential. The cells were cultured in α-minimum essential medium (α-MEM; GIBCO, Invitrogen, Carlsbad, CA, USA) containing 10% fetal bovine serum (FBS; GIBCO, Invitrogen, Carlsbad, CA, USA) and antibiotics (10,000 U mL^–1^ penicillin and 25 μg mL^–1^ streptomycin) (GIBCO, Invitrogen, Carlsbad, CA, USA) under a humidified atmosphere consisting of 5% CO_2_ at 37 °C. The culture medium was changed every 2 or 3 days. For differentiation experiments, the cells were incubated in differentiation medium composed of α-MEM containing 10% FBS, antibiotics, 10 mM β-glycerol phosphate, and 5 μg mL^–1^ ascorbic acid, in the absence (control group) or presence of quercetin, SrQ, or SrR. These compounds were solubilized in DMSO and added to the culture medium (the final strontium concentration was the same for both SrQ and SrR, and the final DMSO concentration in the culture medium was 0.1 vol %) To access the ligand effect, the same pure quercetin or SrQ concentration was added to the medium.

#### 2.4.2 Cytotoxicity/Cell Viability

The ability of the cells to reduce tetrazolium 3-(4,5-dimethylthiazol-2yl)-2,5-diphenyl bromide (MTT, Sigma-Aldrich-USA) was evaluated after culture for 24, 48, and 72 h. For this purpose, a 1 mg mL^–1^ MTT solution was added to the wells after the medium was removed. Next, after incubation at 37 °C for 4 h, the supernatant was removed, the formazan crystals were solubilized in DMSO, and absorbance was measured at 570 nm (SpectraMaxM3). The cell viability results are expressed as percentage relative to the control group (cells cultured in medium containing 0.1% DMSO).

#### 2.4.3 Alkaline Phosphatase Activity Assay

Alkaline phosphatase (ALP) activity was measured as previously reported.^41^ Briefly, the cells were seeded at 2 × 10^4^ cells/well in a 24-well plate and cultured in the presence or absence of the tested complex for 7 or 14 days. Then, the ALP activity was assessed by using *p*-nitrophenyl phosphate as a substrate (*p*-NPP, Sigma-Alrich-USA). The cells were washed twice with phosphate buffer solution (PBS), lysed, and incubated with 3 mM *p*-NPP at 37 °C for 1 h. At the end of incubation, the reaction was stopped by adding NaOH to it, and the absorbance at 410 nm was measured (SpectraMaxM3). The ALP activity was normalized against the total cellular protein, determined as described previously,^42^ and is expressed as U mg^–1^ of total protein content. One unit of enzyme (*U*) is defined as the amount of enzyme hydrolyzing 1.0 nmol substrate at 37 °C per min per mg of protein.

#### 2.4.4 Mineralization Assay

Extracellular matrix mineralization was measured as a late marker of osteogenic differentiation. Briefly, MC3T3 preosteoblasts were seeded in a 24-well plate (2 × 10^4^ cells/well) and treated with quercetin, SrQ, or SrR in osteogenic medium for 14 days. The culture medium was changed every 2 or 3 days. After that, the cells were washed with PBS, fixed with formalin (4 vol %), and stained with alizarin red-S (AR-S; Sigma-Aldrich-USA, pH 4.1) for 10 min. Next, the cell monolayer was washed with PBS three times. For quantitative analysis, acetic acid (10 vol %) was used to solubilize the mineral nodules, and absorbance was measured at 405 nm. Results are expressed as % relative to the control group (cells cultured with osteogenic medium).

### 2.5 In Vivo Experiments

#### 2.5.1 Animal Models

Male isogenic mice of the C57/BL6 lineage, aged between six and 8 weeks, were acquired from the Central Bioterium of the Ribeirão Preto Campus of the University of São Paulo. This research was authorized by the Ethics Committee for the Use of Animals of the Faculty of Pharmaceutical Sciences of Ribeirão Preto of the University of São Paulo under protocol number 23.1.426.60.6.

#### 2.5.2 Apical Periodontitis Induction Model and Treatments

The present study employed the classic protocol for inducing periapical lesions, with some modifications.^43^ Briefly, mice were intraperitoneally anesthetized with ketamine hydrochloride (Ketamine 10%, Agener União Química Farmacêutcia Nacional S/A. Embu-Guaçu, SP) at 0.1 mL/kg of body weight and xylazine hydrochloride (Anasedan 2%, Ceva Santé Animale S/A. Paulnea, SP) at 0.1 mL/kg of body weight. Then, they were positioned on a surgical table for mandibular retraction. The left mandibular first molar was opened by using a stainless-steel ball bur attached to a low-speed handpiece and contra-angle under a stereomicroscope. The pulp was left exposed to the oral environment, and the contralateral tooth was considered to be the control. After the apical lesion model was induced, the mice were divided into two experimental groups. Group 1—control—received vehicle (saline solution) and Group 2 received SrQ (600 μg/kg in 100 μL) intraperitoneally.

The treatment was initiated on the day that the surgery to induce the lesion was accomplished and maintained for 7 days. Subsequently, jaw samples were collected and prepared for microcomputed tomography (μCT) analysis.

#### 2.5.3 Micro-CT Scanning

The mandibles were fixed in 10% neutral buffered formalin for 24 h, which was followed by scanning with a Skyscan 1172X-ray microtomograph (Bruker Corporation, Billerica, MA, USA) with a voxel size of 12 μm, 59 kV, a 0.5 mm aluminum filter, and 0.6° rotation angle. For visualization, three-dimensional projection images were reconstructed from a stack of two-dimensional images; the NRecon software (version 1.6.10; Skyscan; Bruker Corp.) was employed. To determine the lesion volume, three-dimensional analysis was conducted in the first molar root apical region.

### 2.6 Statistical Analysis

All the experiments were performed in triplicate. Statistical analyses were performed by using the software GraphPad Prism version 8.0.1. Analysis of variance followed by Dunnett’s test for simple and multiple comparisons was employed. Values are expressed as the mean ± standard deviation (sd), and significance was set at *p* < 0.05.

## 3 Results and Discussion

### 3.1 SrQ Characterization

**Figure 1:**
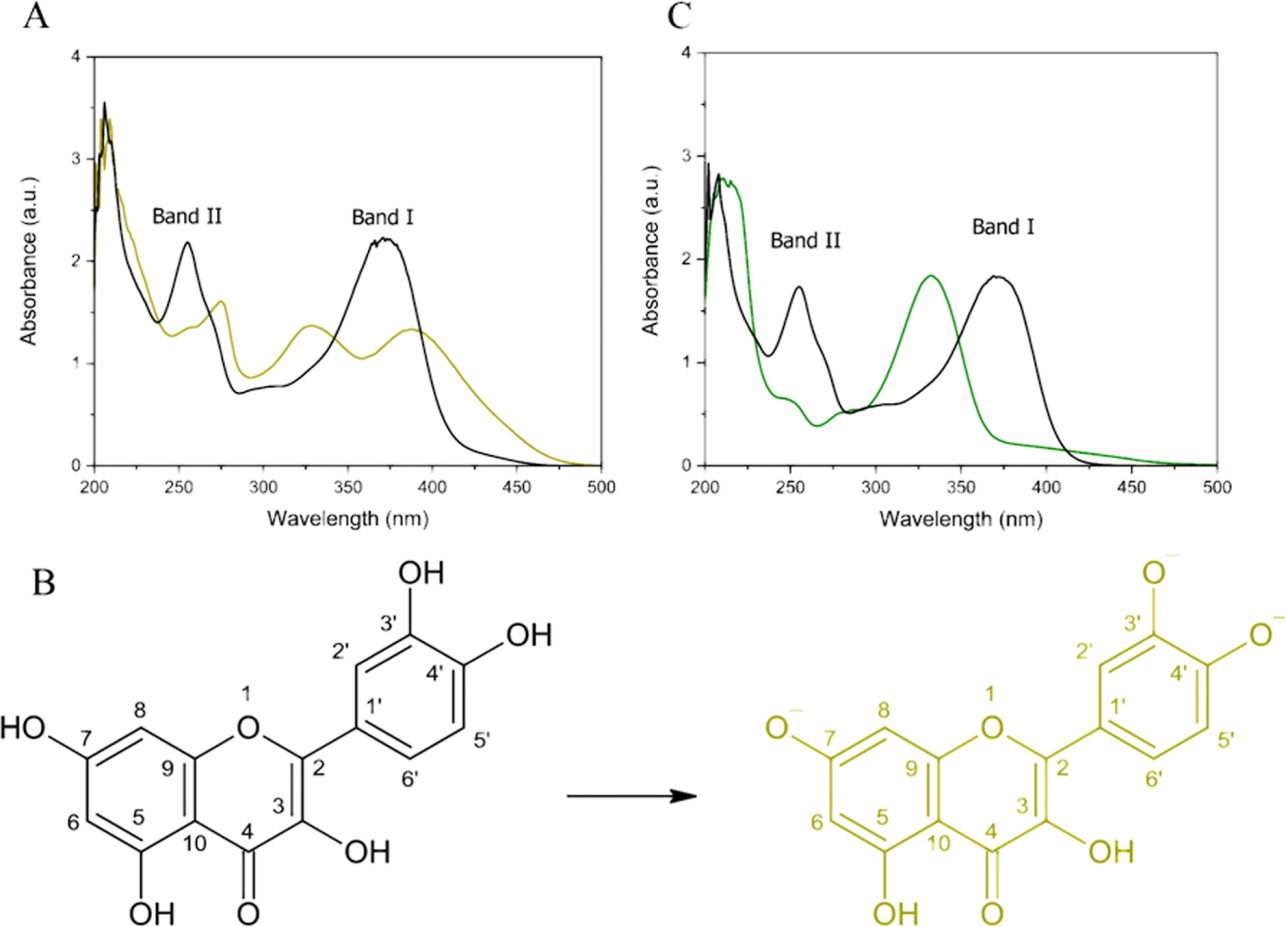
Quercetin and SrQ electronic structures investigated by UV–vis spectroscopy. (A) Quercetin UV–vis absorption spectra in methanol (black line) and basified methanol (yellow line); (B) representation of the quercetin electronic configuration in pure methanol (black structure) and basified methanol (yellow structure); and (C) absorption spectra in the UV–vis region of quercetin (black line) and SrQ (green line) solubilized in methanol (2 × 10^–4^ mol L^–1^).

The quercetin UV–vis spectrum displays two main absorption bands related to the π → π* transitions ranging from 240 to 280 nm (band II) nm, with the maximum peak at 235 nm, and from 300 to 400 nm (band I),^44^ with the maximum peak at 372, as shown in Figure 1A (black line). Band I is associated with electronic transitions in the cinnamoyl group (B ring), whereas band II corresponds to electronic transitions in the benzoyl group. We compared the quercetin UV–vis spectra in methanol and methanol basified with NH_4_OH (Figure 1A) to analyze how the pH we used during the synthesis affects the quercetin structure. In an alkaline medium (yellow line), bands I and II shifted to higher wavelengths because the quercetin OH groups were deprotonated. The bands had lower energy due to the contribution from the electrons, which increased the resonance in deprotonated quercetin. The less intense band at ∼302 nm also shifted to higher wavelength (332 nm) and intensified. Because we synthesized SrQ at pH ≅ 9.00, quercetin groups 3′OH, 4′OH, and 7OH must have been deprotonated during the synthesis, and the bivalent strontium ions must have formed a complex with quercetin through these groups, as shown in the molecular structure represented in Figure 1B.

Strontium complexation with quercetin resulted in a bathochromic shift of the quercetin absorption bands because the conjugative effect increased when SrQ was formed (Figure 1C). These results agree with the data published by Cruz et al., who showed a similar profile for the strontium–morin complex.^20^ The changes in the quercetin electronic profile indicated that the syntheses yielded SrQ.

**Figure 2:**
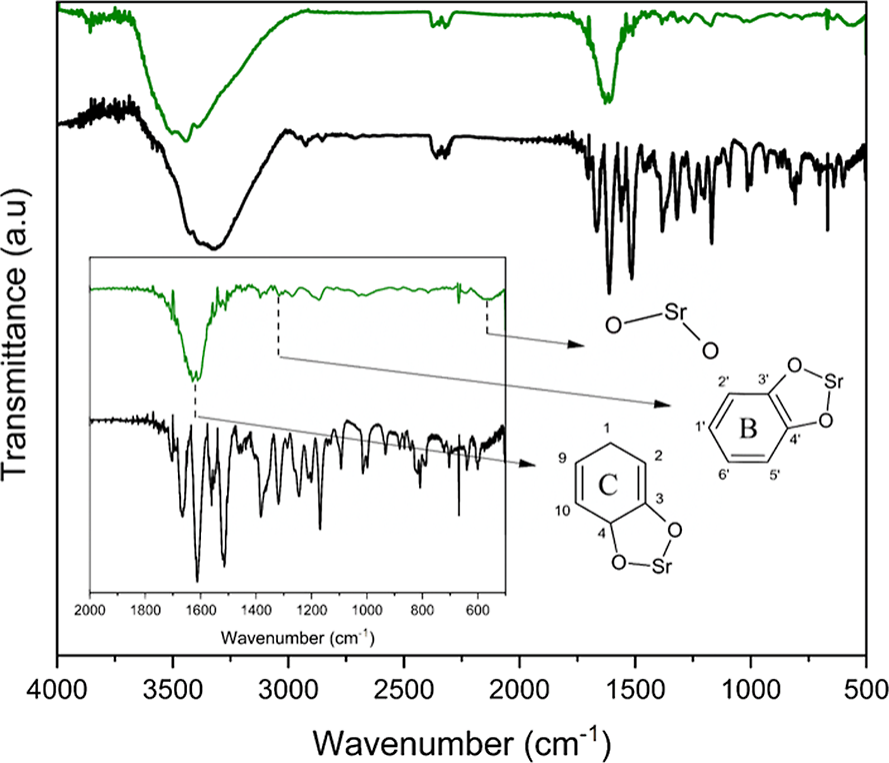
SrQ chemical structure. Quercetin (black line) and SrQ (green line) FTIR spectra. The inset is an amplification of the region from 2000 to 500 cm^–1^, and the black arrows indicate the main bands attesting complexation. ^1^H NMR and elemental analyses were crucial to determine the SrQ stoichiometry. Table 1 lists the chemical shifts obtained for quercetin and SrQ.

We followed the changes in the quercetin chemical structure upon complexation with strontium by Fourier transform infrared (FTIR) analysis. The quercetin FTIR spectrum is complex because it displays bands stemming from vibration of the chemical bonds of aromatic rings, ether group, and carbonyl group between 2000 and 1000 cm^–1^. Compared with the quercetin FTIR spectrum, the SrQ FTIR spectrum had a reduced number of vibrational modes and less intense bands (Figure 2), suggesting that a rigid structure emerges upon quercetin complexation with strontium. Similar data have been reported in studies of quercetin binding to magnesium, copper, or lead.^32,33,45^ Moreover, in the SrQ FTIR spectrum, we identified the bands at 1250 and 1500 cm^–1^, related to ring B and C vibration modes, and the band at 550 cm^–1^, ascribed to O–Sr–O stretching. Together, the UV–vis and FTIR spectra showed that SrQ emerges as the product of the reaction between quercetin and strontium.

**Figure 3:**
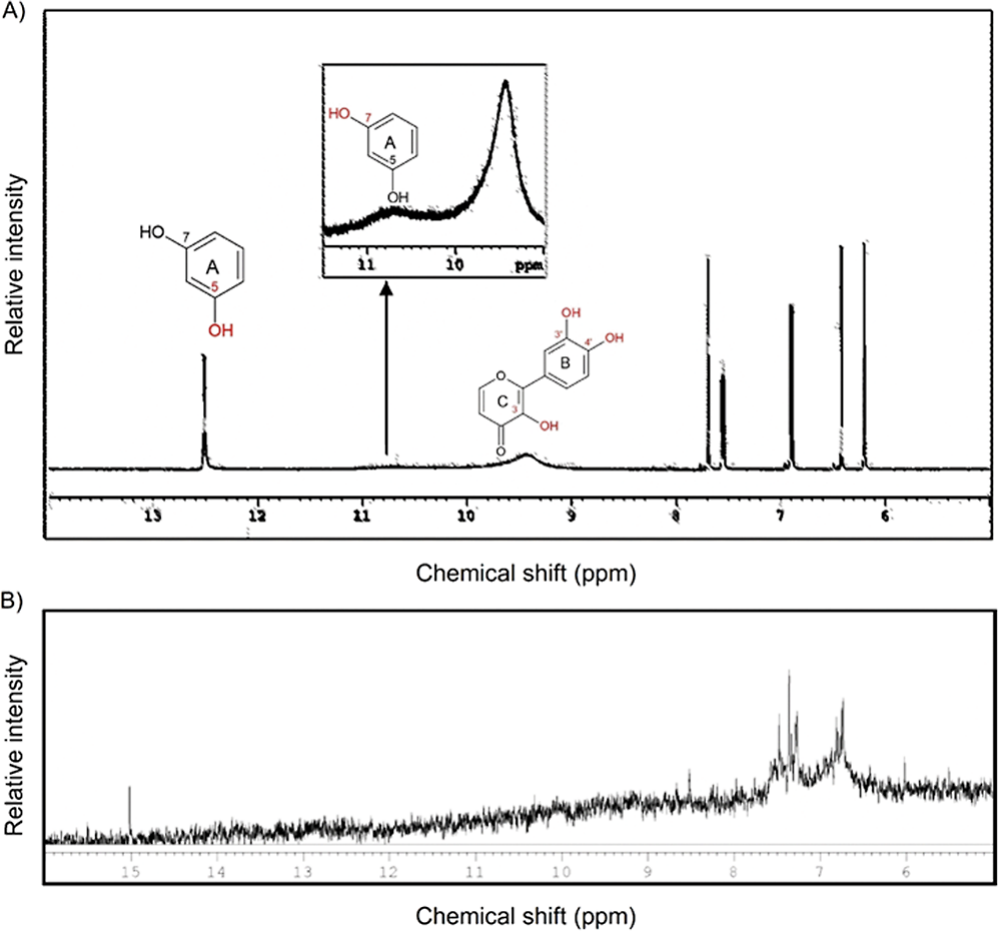
NMR spectra of (A) quercetin and (B) SrQ.

The ^1^H NMR spectra presented in Figure 3 revealed peaks corresponding to the hydrogen atoms of the quercetin 3-OH, 4′–OH, and 3′–OH groups in the quercetin ^1^H NMR spectrum, but these peaks did not appear in the SrQ ^1^H NMR spectrum (Table 1 and Figure 3). The quercetin ^1^H NMR spectrum also displayed a low-intensity peak at 10.72 ppm, related to the 7-OH group hydrogen (see the inset magnification in Figure 3), but this peak was absent from the spectra of the SrQ ^1^H NMR spectrum. On the basis of these results, strontium binds to the quercetin 3′–OH and 4′–OH groups and the 3-OH group of the quercetin C ring carbonyl group. This result agrees with previously reported observations^20,27,29^ of higher acidity, easy deprotonation, and enhanced metal-binding ability of the 3-OH group.

**Table 1:** Chemical Shifts of the Hydroxyl Groups Observed in the Quercetin and SrQ ^1^H NMR Spectra

	δ (ppm)	
group	quercetin	SrQ
5-OH	12.43	15
7-OH	10.72	
3-OH	9.95	
4′-OH		
3′-OH		

We confirmed the SrQ structure by elemental analysis, which indicated the presence of 3.36 wt % hydrogen and 30.96 wt % carbon. This corresponds to 19 hydrogen atoms, seven of which are expected to come from SrQ (Table 2). The 12 other hydrogen atoms should come from the six water molecules bound to the complex. Quantification of 15 carbon atoms, due to the quercetin chemical structure only, validated the SrQ composition. SrQ has an experimental molecular weight of 581.92 g·mol^–1^, which is close enough to the theoretical molecular weight.

**Table 2:** SrQ Elemental Analysis

	carbon (%)		hydrogen (%)		molecular weight (g mol^–1^)	
	theoretical	exp	theoretical	experimental	theoretical	exp
SrQ	30.90	30.96	3.28	3.36	582.39	581.92

Determining the molar conductivity helps determine the charge of a complex. Geary revised the molar conductivity of a series of charged and uncharged complexes.^46^ They found that the quercetin–strontium complex has a conductivity of 162.04 S cm^2^ mol^–1^ in methanol, which indicates that SrQ has 2:1 stoichiometry.^46^

**Figure 4:**
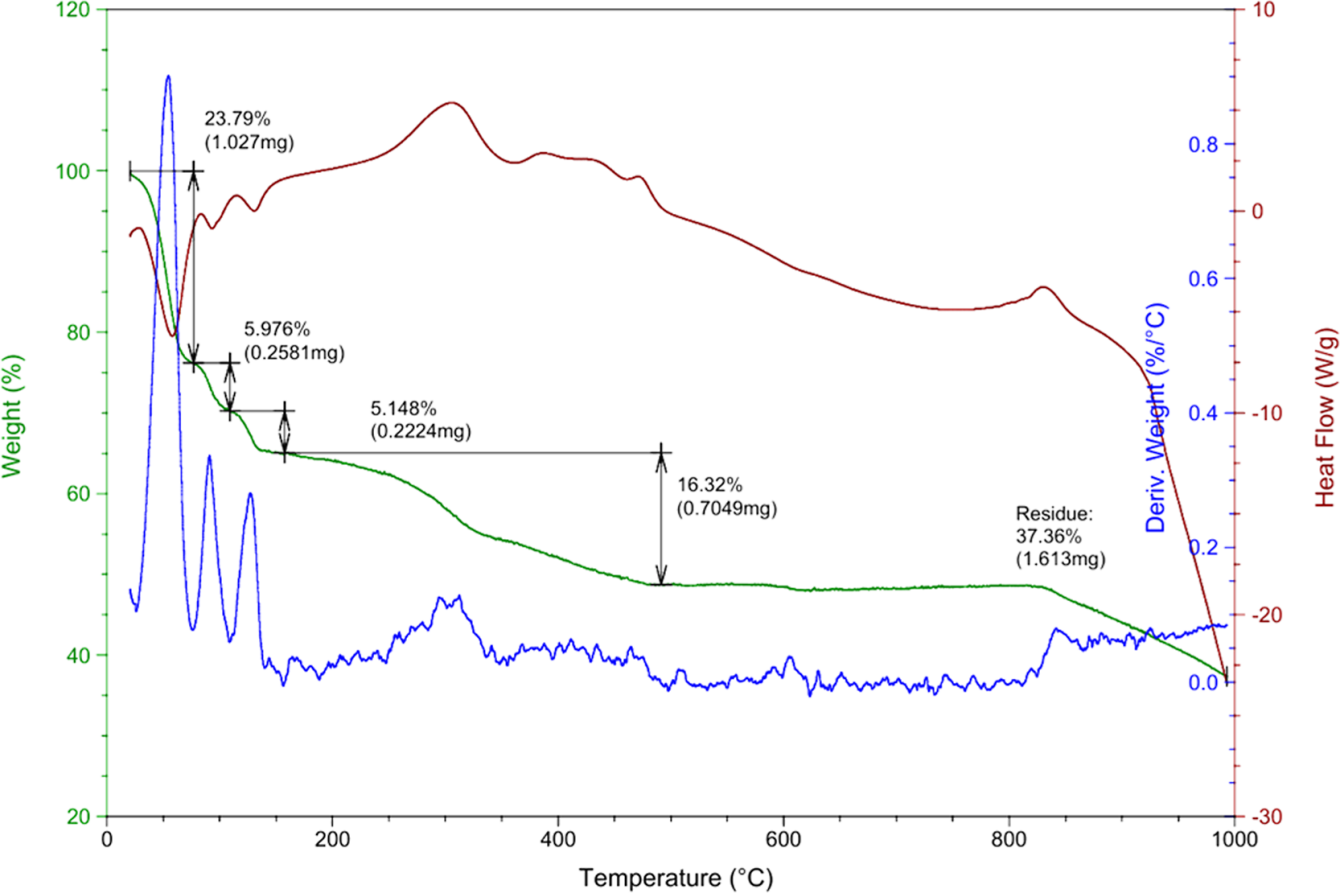
SrQ thermogram in air from 20 to 1000 °C. The blue line represents the first derivative related to mass loss (%/°C), the green line represents the mass loss (%), and the red line concerns the heat flow (W/g).

**Figure 5:**
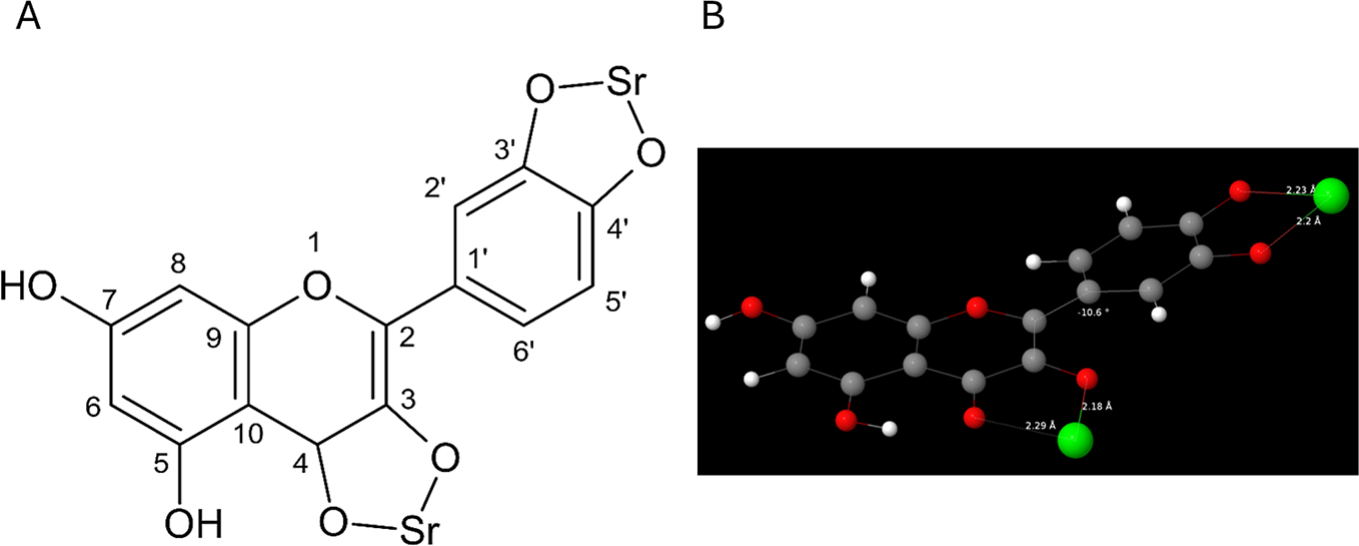
SrQ structure. (A) Suggested SrQ chemical structure on the basis of experimental data; (B) SrQ structure obtained at the B3LYP-D3(BJ)/def2-TZVP level. Gray, red, white, and green spheres correspond to carbon, oxygen, hydrogen, and strontium atoms, respectively.

We accomplished TGA to determine the SrQ stability and to elucidate its structure. The results in Figure 4 revealed that 18.24% mass was lost between 20 and 200 °C. This degradation step refers to the loss of six bound water molecules, as reflected by the endothermic peak of the heat flow curve. The next mass loss step referred to exothermic ligand combustion between 240 and 380 °C, corresponding to 15.67% mass loss. The last step reflected exothermic decomposition to generate metal oxide burning. Considering that the final residue was strontium oxide (SrO; melting point = 2530 °C), it is possible to indicate the number of ions present in SrQ through the molar ratio. Upon analysis of the thermogram (Figure 5), the three initial mass loss steps were related to the loss of adsorbed water and of six water molecules present in the SrQ structure. Bearing in mind that SrQ contains 2 mol Sr^2+^, the final residue must contain 2 mol SrO (strontium oxide). Thus, the initial mass of 4.319 mg of SrQ used during TGA should result in 2.26 mg of SrO. We detected 2.706 mg of residue after total combustion, which also supports the 2:1 stoichiometry of SrQ.

On the basis of spectroscopy, elemental analysis, conductivity measurements, and TGA, we propose the following structure for SrQ: [(C_15_H_7_O_7_)Sr_2_]·6(H_2_O), with a molar weight of 581.92 g·mol^–1^. We show the proposed SrQ molecular structure (water molecules are hidden for simplicity) in Figure 5A.

Theoretical calculations confirmed the SrQ structure. Figure 5B shows the equilibrium geometry. The distances between the quercetin oxygen atoms and the strontium ions are approximately 2.2–2.3 Å. Notably, the quercetin ring B ring is not coplanar with the chromone moiety, with the dihedral angle between these planes being around −11°. Laplacian bond order calculations for each O–Sr pair yielded values of approximately 0.07, indicating that the quercetin oxygen atoms and strontium ions establish an ionic bond. The Gibbs free energy calculations indicated that SrQ is stable (−108 kcal/mol). This stability primarily arose from the Gibbs free energy of the gas-phase reaction,n given that the Gibbs free energy change associated with the transfer from the gas phase to methanol is positive.

### 3.2 In Vitro and In Vivo Experiments

**Figure 6:**
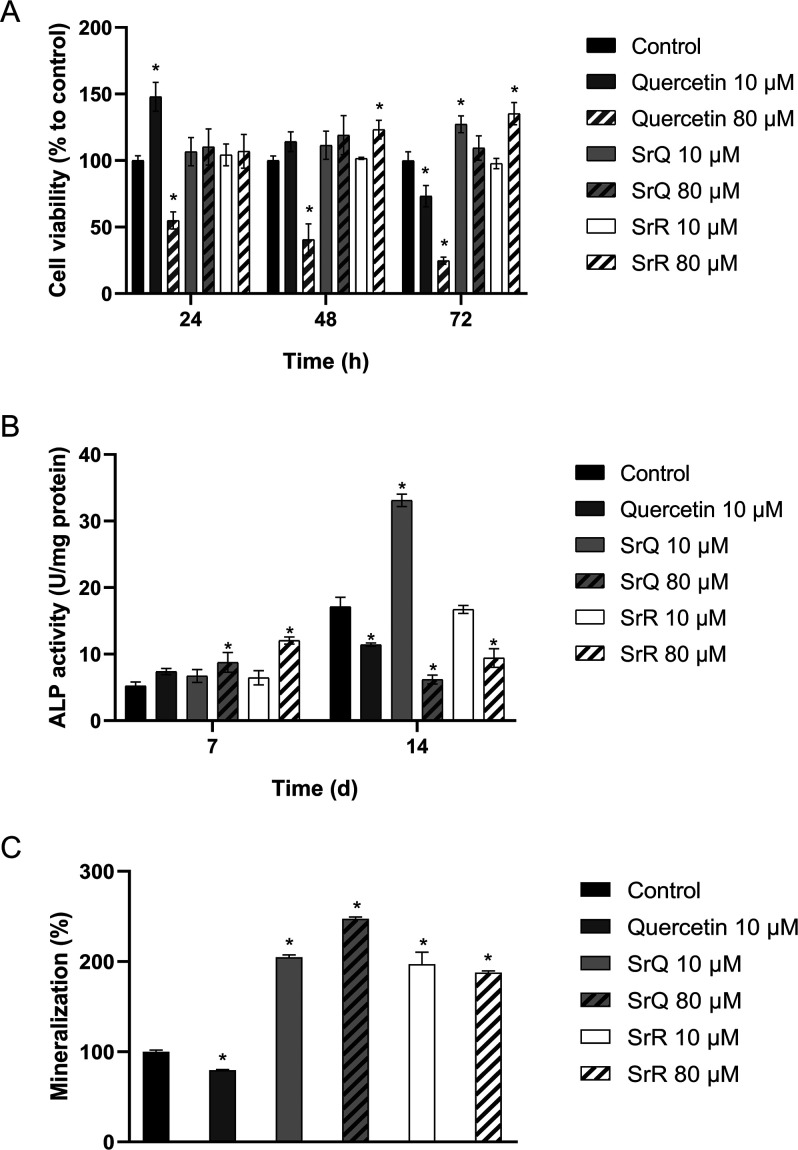
Quercetin, SrQ, and SrR biological effects on MC3T3 preosteoblast cell viability, ALP activity, and mineralization. (A) The cells were treated with 10 or 80 μM quercetin, SrQ, or SrR for 24, 48, or 72 h, and cell viability was measured by the MTT assay. (B) The cells were treated with 10 or 80 μM quercetin, SrQ, or SrR for 7 and 14 days, and ALP activity was assessed by determining *p*-NPP conversion to *p*-nitrophenolate. The results are normalized to the total protein content. (C) The cells were treated with 10 or 80 μM quercetin, SrQ, or SrR for 14 days, and extracellular matrix mineralization was assessed by dissolving mineral nodules by alizarin red staining with acetic acid (10 vol %). Data represent the mean ± sd of three experiments. * = *p* < 0.05 in relation to the control group according to Dunnett’s test.

The biocompatibility of new molecules and materials for medical applications is an important parameter to evaluate when developing new therapies. Biocompatibility can be followed in vitro by means of MTT cytotoxicity assays, which help quantify the mitochondrial activity as a measure of viable cells given that only living cells can reduce MTT to formazan (the colorimetric product for the quantification measurement).^22,50^ Therefore, we determined how quercetin, SrQ, and SrR (10 and 80 μM) affect the MC3T3-E1 viability. We used SrR for comparison purposes because its positive effect on in vitro mineralization by osteoblasts has been described.^13,47,51,52^ The results shown in Figure 6A revealed that, compared to the control, higher quercetin concentration reduced cell viability by 45, 59, and 75% after culture for 24, 48, and 72 h (*p* < 0.05), whereas lower quercetin concentration induced transient proliferation after 24 h (*p* < 0.05). In agreement with our results, quercetin at concentrations higher than 50 μM has been shown to reduce the viability of a breast cancer cell line (MCF-7) by inducing apoptosis and necrosis.^53^ Another study has shown that quercetin at concentrations between 50 and 100 μM reduces the viability of mouse bone mesenchymal stem cells after culture for 24 h.^23^ Interestingly, complexing strontium with quercetin reduced its cytotoxicity when compared to pure quercetin at all the investigated time points. This is an interesting result for SrQ biomedical applications, indicating that higher quercetin doses can be delivered to the cell without eliciting any cytotoxicity when strontium is complexed with quercetin. In accordance with our findings, no cytotoxicity was found in the case of MC3T3-E1 cells cultivated in the presence of a morin–strontium complex at the same strontium and ligand concentrations used herein for 72 h.^20^ Due to its higher cytotoxicity, we excluded the 80 μM quercetin group from further differentiation assays.

The observation that 10 μM quercetin promotes cell proliferation at 24 h but reduces it at 72 h suggests a time-dependent effect. Initially, quercetin may enhance cell growth through its antioxidant properties,^22,23^ but prolonged exposure may induce cytotoxicity or oxidative stress. This biphasic response is common with quercetin, where low doses stimulate proliferation,^22^ but higher or extended exposure leads to reduced cell viability.^53^ Further investigation into the long-term effects of quercetin is needed to assess the potential cytotoxicity.

ALP activity is an early marker of osteoblast differentiation and is frequently used when studying osteogenic differentiation.^54^ Therefore, we determined the ALP activity in MC3T3-E1 preosteoblasts treated with quercetin, SrQ, or SrR for 7 or 14 days. The ALP activity increased significantly (about 67.3%) in the cells treated with 80 μM SrQ for 7 days (*p* < 0.05, Figure 6B). Surprisingly, the ALP activity was higher in the cells treated with 10 μM SrQ than in those treated with 80 μM SrQ for 14 days (*p* < 0.05; Figure 6B). We observed the same trend for the cells treated with SrR. The ALP activity depends on the optimal concentration of divalent ions and decays with higher concentrations,^55^ which might explain the results. This finding also agrees with the findings of Cruz et al., who showed higher ALP activity for preosteoblasts treated with 80 μM morin–strontium for 7 days and 10 μM morin–strontium for 14 days.^20^

Extracellular matrix mineralization is the last stage of osteogenic differentiation.^4^ Therefore, we evaluated mineralization of MC3T3 preosteoblasts cells continuously treated with quercetin, SrQ, or SrR for 14 days by alizarin red-S staining (results are displayed in Figure 6C). Compared to the control, mineralization was enhanced by 2- and 2.5-fold when the cells were treated with 10 and 80 μM SrQ, respectively (*p* < 0.05). Quercetin at 10 μM and SrR at 10 or 80 μM decreased and enhanced mineralization by 2- and 1.8-fold, respectively, compared to the control (*p* < 0.05). Cruz and collaborators described that extracellular mineralization increases slightly in MC3T3 cells continuously treated with strontium–morin over 21 days. In contrast, our data showed that SrQ performs better, with a higher quantity of mineral nodules being formed after culture for 14 days when we used the same strontium complex concentrations employed by Cruz et al. for 21 days.^20^

Other metal-flavonoid complexes have been studied, aiming at applications in osteogenesis. For instance, copper and magnesium complexes with quercetin have been shown to possess antioxidant and anti-inflammatory properties, which are beneficial in bone tissue regeneration.^35,36^ Similarly, strontium has been highlighted as a key player in bone health due to its ability to promote osteoblast differentiation and enhance mineralization, potentially offering advantages over other metals, like calcium or zinc, in terms of bone strength and density.^9,47^ Furthermore, flavonoids such as kaempferol and myricetin exhibit similar synergistic effects when complexed to metals, though their interactions differ compared to quercetin due to the different chemical structures that influence the stability and bioactivity of the complex.^48,49^ These comparative studies underline the unique role of strontium in combination with quercetin, suggesting that the SrQ complex could have distinct advantages for osteogenic applications.

**Figure 7:**
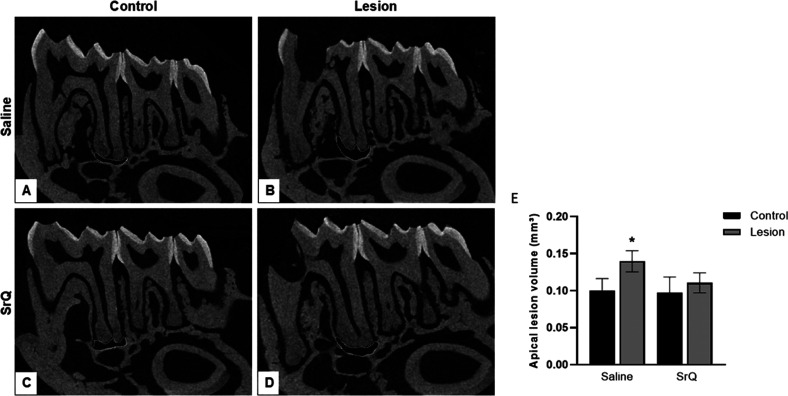
Effect of treatment with SrQ on apical lesion formation in an experimental model. (A–D) Representative image of the micro-CT section obtained from saline- or SrQ-treated mice (600 μg/kg) after exposure of the pulp. (E) Periapical lesion volume (mm^3^) as measured in the root apex of all the groups (yellow arrows show delimitation). Values of lesion volume are expressed as mean ± sd. * = *p* < 0.05 in relation to the control group treated with saline, according to Dunnett’s test.

To validate the biological relevance of SrQ in bone remodeling in vivo, we investigated whether treatment with SrQ influences pathological bone remodeling. For this purpose, we induced bone loss in a pathological chronic inflammatory condition by employing the apical periodontitis model.^43^ Briefly, exposing the dental pulp to the oral microenvironment led to chronic inflammatory lesions characterized by increased lesion size in the root periapical region (Figure 7A,B). Micro-CT imaging showed that submitting mice with induced periodontitis to treatment with SrQ for 7 days prevented lesions from developing so that periapical lesions were smaller than in the control group (Figures 7C–E).

The fact that the injury did not progress in the animal model with periapical lesions treated with SrQ compared to the control highlighted that this complex is a promising therapeutic option to treat bone loss. The beneficial effect of quercetin concentrations lower than 10 μM on osteogenic differentiation has been described in vitro.^23−26,37,38^ Quercetin at 10 μM has been described to promote osteogenic differentiation of rat mesenchymal stem cells by increasing the ALP activity by about 98% over three.^38^ Kim and collaborators evaluated how three different flavonols (quercetin, kaempferol, and chrysin) affect human adipose stromal cell differentiation by using an osteoblast phenotype, and they obtained better results for 5 μM quercetin, which increased extracellular matrix mineralization by about 150% after treatment for 14 days.^37^ Additionally, quercetin concentrations up to 5 μM have been shown to promote osteogenic cell differentiation and antioxidant responses from mouse bone mesenchymal stem cells through activation of the AMPK/SIRT1 signaling pathway.^23^ Herein, we have shown that SrQ enhances preosteoblast cell differentiation by increasing ALP activity and extracellular matrix mineralization. Moreover, SrQ has other benefits, such as anti-inflammatory and antioxidant responses due to the presence of quercetin, as well as a positive effect on osteoblast maturation due to the strontium osteophytic property.^56^ Furthermore, strontium contributes with an antibacterial feature, as described by some studies,^22,57,58^ which is important for medical applications. Here, we have shown that SrQ is biologically relevant in vivo because it inhibits bone loss in an experimental model. Although the mechanism underlying this protective effect is not clear, our results indicate that SrQ can be a potent platform to induce osteoblast mineralization at low doses compared to SrR, which is the current drug option for treating osteoporosis. Finally, using the quercetin and strontium complex could be optimized to achieve quercetin antioxidant and anti-inflammatory modulation properties together with strontium osteogenic property, which should be evaluated in further studies.

Despite the promising results obtained from physical chemical characterization and in vitro studies, some limitations should be considered in this study. First, the in vivo experiments were conducted in a single animal model, which may not fully represent the complex biological conditions of bone loss or other diseases in humans. Further validation in other animal models, particularly those that simulate human conditions more closely, would strengthen the conclusions. Second, the mechanism of action underlying the osteogenic effects of the SrQ complex remains unclear. While we observed promising results in terms of ALP activity and mineralization, future studies are needed to explore the molecular pathways involved. Additionally, while the quercetin–strontium complex showed promising biocompatibility and efficacy in vitro and in vivo, the potential long-term effects and safety of chronic exposure to this complex remain unexplored. Finally, the study primarily focuses on the effects of SrQ in bone remodeling and mineralization, but its interactions with other physiological systems, tissues, or bone coculture models have not been investigated. Therefore, future studies should address these aspects to fully understand the therapeutic potential and limitations of SrQ.

## 4 Conclusions

We reacted quercetin and strontium in basic medium to obtain a new complex that we chemically characterized as [(C_15_H_7_O_7_)Sr_2_]·6(H_2_O). Next, we demonstrated that this new complex is beneficial for in vitro osteogenic differentiation—it increases ALP activity and extracellular matrix mineralization in preosteoblast cells. We then realized the potential in vivo relevance of this new complex by verifying that it can inhibit bone loss in an experimental model, which makes it a candidate drug in bone regenerative medicine. The complex can be incorporated into biomaterials aiming at bone regeneration, providing a novel approach for enhancing bone repair and overall skeletal health.

In the future, the mechanism of action involved in the osteogenesis promoted by the complex will be investigated to propose a new treatment approach. This investigation will provide a deeper understanding of how the complex exerts its effects at the molecular level, potentially leading to the development of more targeted and effective therapeutic strategies for bone regeneration.
